# LINC00174 Facilitates Proliferation and Migration of Colorectal Cancer Cells via MiR-3127-5p/ E2F7 Axis

**DOI:** 10.4014/jmb.2103.03001

**Published:** 2021-06-14

**Authors:** Yuhong Ma, Yuzhen Li, Yuanyuan Tang, Ning Tang, Dengke Wang, Xiaofei Li

**Affiliations:** 1 Department of Gastroenterology, People’s Hospital of Ningxia Hui Autonomous Region, Jinfeng District, Yinchuan City, Ningxia Hui Autonomous Region 750021, P.R. China; 2 Department of Anatomy, Ningxia Medical University, Xingqing District, Yinchuan City, Ningxia Hui Autonomous Region 750003, P.R. China

**Keywords:** LINC00174, colorectal cancer, migration, miR-3127-5p, E2F7, epithelial-mesenchymal transition

## Abstract

The literature indicates that LINC00174 promotes the growth of colorectal cancer (CRC) cells, but its research needs to be enriched. We tried to explore the function and mechanism of LINC00174 in CRC cell proliferation and migration. Bioinformatics analysis predicted the binding relationship and expressions of lncRNA, miRNA and mRNA. Clinical study analyzes the relationship between LINC00174 and clinical data characteristics of CRC patients. The expressions of LINC00174, miR-3127-5p and E2F7 were verified by RT-qPCR, and the combination of the two was verified by dual luciferase analysis and RNA immunoprecipitation as needed. Western blot was used to detect the expression of EMT-related protein and E2F7 protein. Functional experiments were used to evaluate the function of the target gene on CRC cells. LINC00174 was up-regulated in CRC clinical samples and cells and was related to the clinical characteristics of CRC patients. High-expression of LINC00174, contrary to the effect of siLINC00174, promoted cell viability, proliferation, migration and invasion, up-regulated the expressions of N-Cadherin, Vimentin, E2F7, and inhibited the expression of E-Cadherin. MiR-3127-5p was one of the targeted miRNAs of LINC00174 and was down-regulated in CRC samples. In addition, miR-3127-5p mimic partially reversed the malignant phenotype of CRC cells induced by LINC00174. Besides, E2F7 was a target gene of miR-3127-5p, and LINC00174 repressed miR-3127-5p to regulate E2F7. Our research reveals that LINC00174 affected the biological characteristics of CRC cells through regulated miR-3127-5p/ E2F7 axis.

## Introduction

Colorectal cancer (CRC) is a common cancer in the human digestive tract [1]. Tumor growth and metastasis are the main causes of death in CRC patients, especially in patients with advanced CRC [2]. In recent years, there has been great development in the treatment of CRC, but there are still many challenges [3, 4]. Therefore, the determination of CRC biomarkers has very special significance for improvement of prognosis [5].

Long non-coding RNAs (lncRNAs) play a meaningful role in the occurrence of CRC and can be used as a marker for CRC diagnosis [6]. Yin *et al*. reported that the low expression of MEG3 was markedly associated with tumor histological grade and lymph node metastasis, while MEG3 overexpression significantly inhibited colorectal cancer cell proliferation [7]. H19 is high-expressed in CRC tissues, and overexpression of H19 can substantially promote epithelial-mesenchymal transition (EMT) and accelerate tumor growth in vivo [8]. Ding *et al*. found that loc554202 was remarkably down-regulated in CRC cell and tissues, and regulated apoptosis through the activation of specific protease cleavage cascades [9]. LINC00174 has been shown to as an oncogenic lncRNA in a variety of cancer [10-14], for example, LINC00174 promotes glycolysis and tumor progression by regulating miR-152-3p/SLC2A1 axis in glioma [10]; Silencing of LINC00174 decreases chemoresistance to temozolomide in human glioma cells by regulating miR-138-5p/SOX9 axis [11]. LINC00174 functions as a sponge for miR-320, increase the expression level of oncogene S100A10 in HCC, LINC00174 promotes the tumorigenesis and progression of hepatocellular carcinoma [13]; Promotion of BZW2 by LINC00174 through miR-4500 inhibition enhances proliferation and apoptosis evasion in laryngeal papillomathymic epithelial tumors [15]. In addition, there are literatures indicating that silencing LINC00174 can inhibit the growth of CRC cells by regulating miR-1910-3p/TAZ axis [16], but the function and molecular regulatory network of LINC00174 in CRC need to be improved.

Understanding the mechanism of lncRNAs and their target genes has gradually become the focus of academic study [17]. LncRNAs participate in tumor progression through multiple mechanisms [18, 19]. For example, CRNDE affects the progress of CRC through miR-181a-5p acting on β-catenin and TCF4 [20]. LncRNA H19 regulates the biological characteristics and EMT process of CRC cells through the miR-29b-3p/PGRN/Wnt axis [21]. The above studies indicate that lncRNA-miRNA interaction plays a key role in CRC.

In addition, we obtained 7 miRNAs that bind to LINC00174 and are lowly expressed in rectal adenocarcinoma through bioinformatics analysis. Among them, miR-3127-5p has been rarely studied in diseases, and so far there have been only two reports. MiR-3127-5p was found to play a role in suppressing cancer in non-small-cell lung cancer (NSCLC) [22]. Moreover, as the target miRNA of lncRNA GACAT3, miR-3127-5p affects the progression of glioma by regulating the expression of ELAVL1 [23]. Although the latest research shows that miR-3127-5p is differentially expressed in CRC, its function and mechanism are still unclear [24]. Thus, we constructed clinical and cell assays accompanying bioinformatics analysis to investigate whether LINC00174 affects the biological process of CRC cells by regulating miR-3127-5p.

## Materials and Methods

### Ethics Statement and Tissue

Prior to this study, all patients were informed and signed informed consent. The clinical specimens were obtained from 80 CRC patients who came to our hospital from June 2019 to April 2020, after approval of the ethics committee of People’s Hospital of Ningxia Hui Autonomous Region (CR201905020). The relationship between LINC00174 and clinical data characteristics of 80 CRC patients were shown in Table 1.

### Bioinformatics Prediction

Target miRNAs of LINC00174 were predicted by starBase v2.0 website (http://starbase.sysu.edu.cn/starbase2/). The miRWalk (http://mirwalk.umm.uni-heidelberg.de/), miRDB (http://mirdb.org/), starBase, Targetscan (http://www.targetscan.org/vert_72/docs/help.html) were used to predict miR-3127-5p target genes. The Cancer Genome Atlas (TCGA) database (http://cancergenome.nih.gov/) analyzed miRNAs or mRNAs that are abnormal expressed in rectal adenocarcinoma. Venn diagram (Venny 2.1.0 website) was used to analyze the intersection of the data generated from the databases.

### Cell and Culture

CCD-18Co cell lines (CRL-1459, normal) and LS174T cell lines (CL-188, tumor) procured by American Type Culture Collection (ATCC, USA), were cultivated in EMEM (30-2003, ATCC, USA) containing 10 % Fetal Bovine Serum (FBS) (30-2020, ATCC) in 37°C, 5% CO_2_. HCT116 (CCL-247), HCT-15 (CCL-225), SW480 (CCL-228), LOVO (CCL-229) cell lines and the corresponding medium were also purchased from ATCC. LOVO cells were cultured in F-12K Medium (30-2004); HCT116 cells were cultivated in McCoy's 5A Medium (30-2007); HCT-15 cells were cultivated in RPMI-1640 Medium (30-200); SW480 cells were cultivated in Leibovitz's L-15 Medium (30-2008).

### Cell Transfection

To overexpress LINC00174, SW480 or LOVO cells were transfected with pcDNA3.1-LINC00174 plasmid using Lipofectamine 3000 (L3000015, Invitrogen, USA) refer to the instructions. The pcDNA3.1 blank plasmid (V79520, Invitrogen) was served as NC group. For the silencing of LINC00174, shLINC00174 plasmids (target sequence: TCTCCAGGTCGTTTCTCCCTGTCTT, GenePharma Company, China) was transfected into SW480 or LOVO cells using Lipofectamine 3000. MiR-3127-5p mimics (miR10014990-1-5) and mimic control (miR1N0000001-1-5) were obtained from Guangzhou RIBOBIO Biotechnology Co. LTD (China). When the confluence of SW480 or LOVO cells in a 24-well plate reached 70-90%, they were ready for transfection.

### Real Time Quantitative Polymerase Chain Reaction

RNAiso Plus (9108Q, Takara, China) or RNAiso for Small RNA (9753Q) were used to isolate total RNA or miRNA, as required. Reverse transcription step was refered to the instructions of PrimeScrip RT reagent Kit (RR037Q) produced by Takara, China. The RT-qPCR detection was constructed in the Mx3000P Real-Time QPCR System (Agilent, USA) with Takara TB Green Premix Ex Taq II (RR820Q, China), followed by the conditions set as below: pre-denaturation (95°C, 3 min), denaturation (95°C, 15 s) and annealing (60°C, 1 min) for a total of 40 cycles, and finally extended (68°C, 7 min). GAPDH or U6 were used as control for mRNA or miRNA, and the results were quantified in the form of 2^-ΔΔCt^ method [16]. Sequences of the primers were listed as follows (5’-3’). LINC00174: (GGCCCAACACTTCCCTCAAA, CAGGGAGAAACGACCTGGAG); miR-3127-5p:(ATCAGGGCTTGTGGAATGGG, GTATCCAGTGCGTGTCGTGG); E2F7: (AAAGGGACTATTCCGACCCAT, ACTTGGATAGCGAGCTAGAAACT); SHBG: (GCCCAGGACAAGAGCCTATC, CCTTAGGGTTGGTAT CCCCATAA); GAPDH: (GCAAGAGCACAAGAGGAAGA, ACTGTGAGGAGGGGAGATTC); U6: (CTC GCTTCGGCAGCACA, AACGCTTCACGAATTTGCGT).

### Cell Counting Kit (CCK)-8

After transfected for 24 h, SW480 or LOVO cells were replaced with fresh medium to continue culturing. Cells in good growth condition was taken to make a single cell suspension (1 × 10^4^ cells/ml) and seeded in 96-well plates. When SW480 or LOVO cells were cultured for 24, 48, or 72 h, 10 μl of CCK-8 reagent (C0037, Beyotime, China) was added to each well for routine incubation for 1 h. Finally, iMark microplate reader from Bio-Rad (USA) was applied to detect the optical density value (OD450) wavelength of each well to indicate cell activity.

### Cell Colony Formation Assay

Consistent with the above experiment, first, each group of cells was adjusted to a cell suspension (1 × 10^4^ cells/ml) and routinely cultivated in a 6-well plate. At about 14 days of culture, macroscopic cell colonies grew in the culture plate. Next, each well was fixed with 4% paraformaldehyde for 15 min, and Giemsa staining reagent (32884, Sigma, USA) was added for staining for 15-20 min. Finally, the optical microscope (DMi8, Leica, Germany) took pictures to record the cell proliferation.

### Wound Healing Assay

The pre-digested CRC cells (1 × 10^5^ cells/ml) were added into ibidi cell plugin (ibidi, 81176, Germany) routinely incubated for 24 h. Next, the ibidi wound healing insert was raised with tweezers. Microscopic examination (CKX53, Olympus, Japan) was performed on the cells grown the day before (Magnification × 100).

### Transwell

Matrix gel (354230, BD Biosciences, USA) was applied to the bottom membrane of the Transwell chamber (8 μm, BD Biosciences). Also, the Transwell chamber was inserted into the 24-well plate. 50 μl cell suspensions were seeded in the upper chamber, whilst RPMI-1640 medium with 10% FBS was added in lower chamber. After cells were cultured for 24 h, the chamber was removed. Invaded cells were fixed and then stained with 0.5% crystal violet for 30 min. Finally, five fields of the cells were randomly observed under the microscope to calculate the number of cell invasion (Magnification × 250).

### Target Gene Verification

The wild-type or mutant sequences of LINC00174 or E2F7 were integrated into the pmirGLO vector (E1330, Promega, USA). Different recombinant plasmids together with miR-3127-5p (M) or mimic control (Blank) were transfected into SW480 or LOVO cells, respectively. Luciferase activities were measured in dual luciferase system (D0010-100T, Solarbio, China) by GloMax 20/20 detector (Promega). Imprint RNA Immunoprecipitation Kit (RIP, Sigma-Aldrich) was constructed to RNA immunoprecipitation (RIP) assays. Briefly, CRC cells were lysed using RIR lysisi buffer, and protein A/G beads conjugated with anti-Argonaute2 (Ago 2) was added to cell extract for 6 h at 4°C. After that, samples were incubated with roteinase K to isolate RNA-protein complexes. IgG was served as control. Finally, immunoprecipitated RNA was subjected to RT-qPCR detection.

### Western Blot

The proteins in this study were lysed using RIPA Lysis Buffer (C05-01001, Bioss, China), followed by quantified with BCA Kit (A53225, ThermoFisher, USA). 20 μg of protein was taken to perform electrophoresis with SDS-PAGE, and then transferred to PVDF membrane (IPVH00010, Millipore, USA). After sealing with blocking Buffer (37565, Thermo Scientific) for 2 h, membranes were then incubated with primary antibodies (4°C, overnight). Afterwards, membranes were incubated with secondary antibodies. Protein bands were visualized with ECL luminous fluid (WBKlS0100, Millipore) and analyzed with gel imaging system (Tanon 2500, Solarbio, China) and Quantity One image analysis software (Bio-Rad, USA). All antibodies produced by Abcam (UK) were as follows: E-Cadherin (ab40772, 1/10000, 97 kDa); N-Cadherin (ab18203, 1/10000, 130 kDa); Vimentin (ab92547, 1/1000, 54 kDa); E2F7 (24489-1-AP, 1/1000, 90 kDa, Proteintech, USA); GAPDH (ab8245, 1/5000, 36 kDa); Goat Anti-Rabbit (ab205718); Goat Anti-Mouse (ab205719).

### Statistical Analysis

**Fig. 1:**
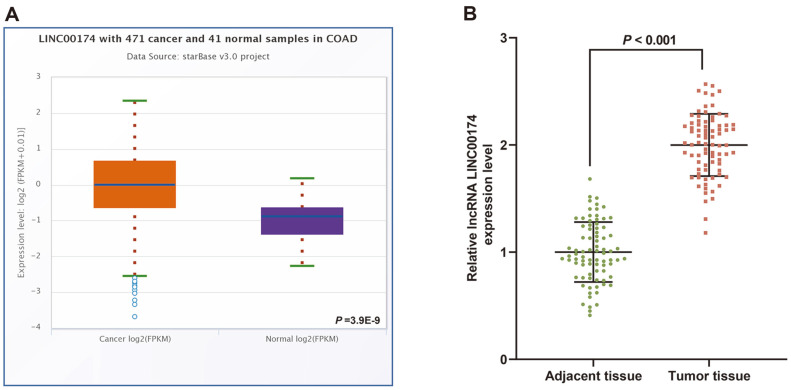
Expression of LINC00174 in colorectal cancer (CRC). (**A**) The starBase website (http://starbase.sysu.edu.cn/starbase2/ ) was used to predict the expression of LINC00174 in colon adenocarcinoma (COAD). Cancer samples = 471, Normal samples = 41. *P* = 3.9E-9. (**B**) The expression of LINC00174 in cancer tissues and adjacent tissues of CRC patients (N = 80) was detected by real-time quantitative PCR (RT-qPCR).

Data were described by mean ± SD; comparison between count data groups was analyzed using χ^2^ test. The data of Fig. 1B used paired sample *t* test; one-way ANOVA for comparison between multiple groups; Tukey test for pairwise comparison between groups. All statistical analysis were implemented using Graphpad 8.0 software, with *p* < 0.05 considered as significant.

## Results

### LINC00174 Was Up-Regulated in CRC and Was Associated with Poor Prognosis

**Fig. 2:**
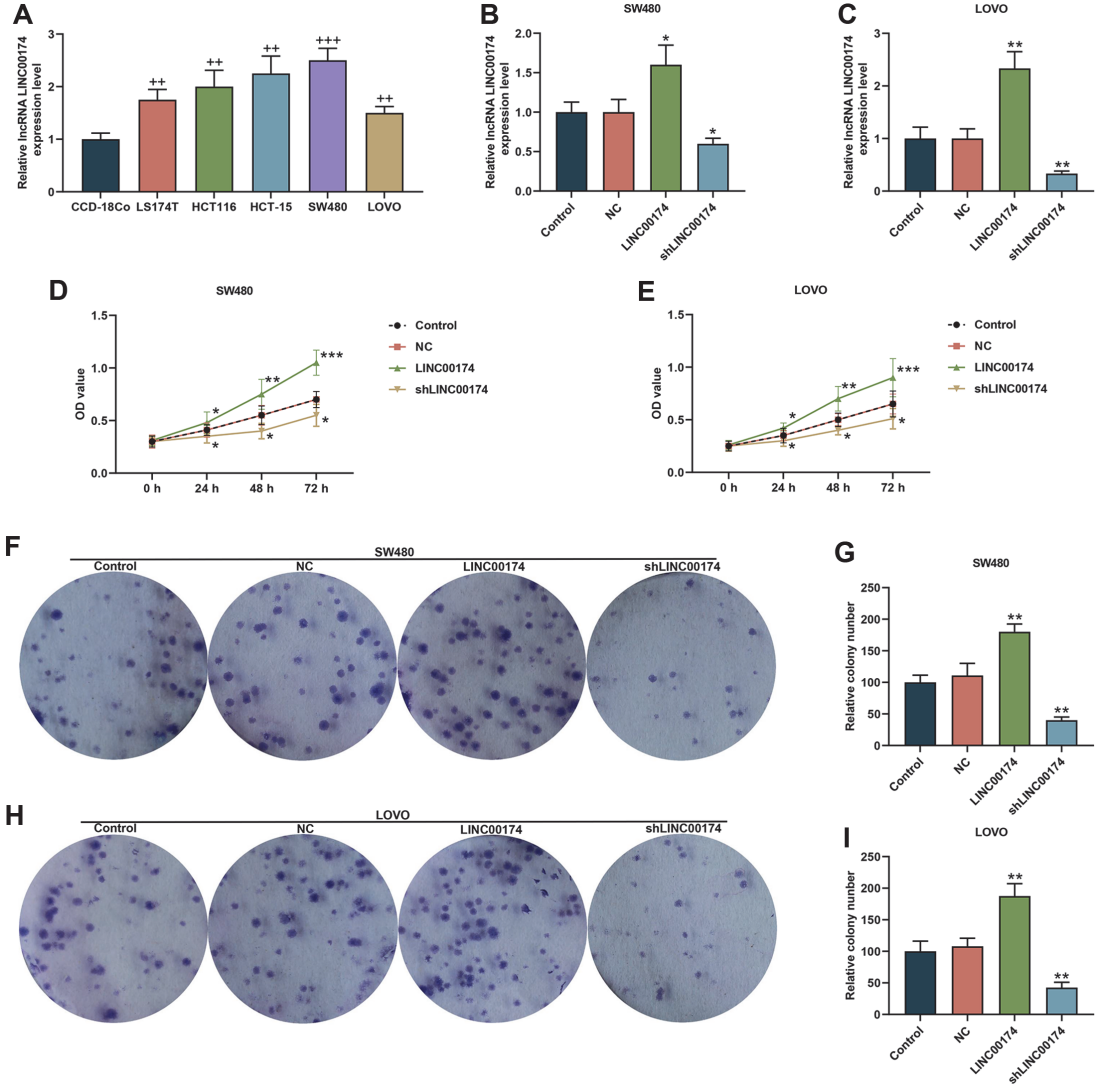
LINC00174 modulated the viability and proliferation of colorectal cancer (CRC) cells. (**A**) The expression of LINC00174 in human normal colon cell line and CRC cell line was detected by RT-qPCR. (**B-C**) The transfection rate of SW480 or LOVO cells transfected with LINC00174 overexpression or shLINC00174 was detected by RT-qPCR. (**D-E**) High expression of LINC00174 promoted cell viability; low expression of LINC00174 had done the opposite, as determined by Cell Counting Kit (CCK)-8. (**F-I**) Clone formation assay was used to test the effect of LINC00174 overexpression or knockdown on SW480 or LOVO cell proliferation. ^++^*p* < 0.01, ^+++^*p* < 0.001 vs CCD-18Co; *p < 0.05, **p < 0.01, ***p < 0.001 vs Negative Control (NC).

Fig. 1A showed LINC00174 was highly expressed in colon adenocarcinoma (COAD) (*P* = 3.9E-9). Based on this, we collected 80 clinical samples of CRC patients, and found that the level of LINC00174 was elevated in tumor tissue (*p* < 0.001, Fig. 1B). In addition, we assessed the relationship between LINC00174 and the clinical data characteristics of CRC patients, and found that the level of LINC00174 was memorably associated with tumor size, lymphatic vessel invasion, tumor invasion depth, lymph node metastasis, distant metastasis and tumor stage (*p* < 0.05, Table 1). We further examined the situation of LINC00174 in the CRC cell line and found that the expression of LINC00174 in LS174T, HCT116, HCT-15, SW480, LOVO cells was notably higher than that of CCD-18Co cells (*p* < 0.01, Fig. 2A). Among all CRC cell lines, the expression of LINC00174 was highest in SW480 cells and lowest in LOVO cells (*p* < 0.01, Fig. 2A), so these two cell lines were selected as the next experimental cells.

### Overexpression of LINC00174 Promoted the Biological Function of CRC Cells, while Silencing LINC00174 Was the Opposite

**Fig. 3:**
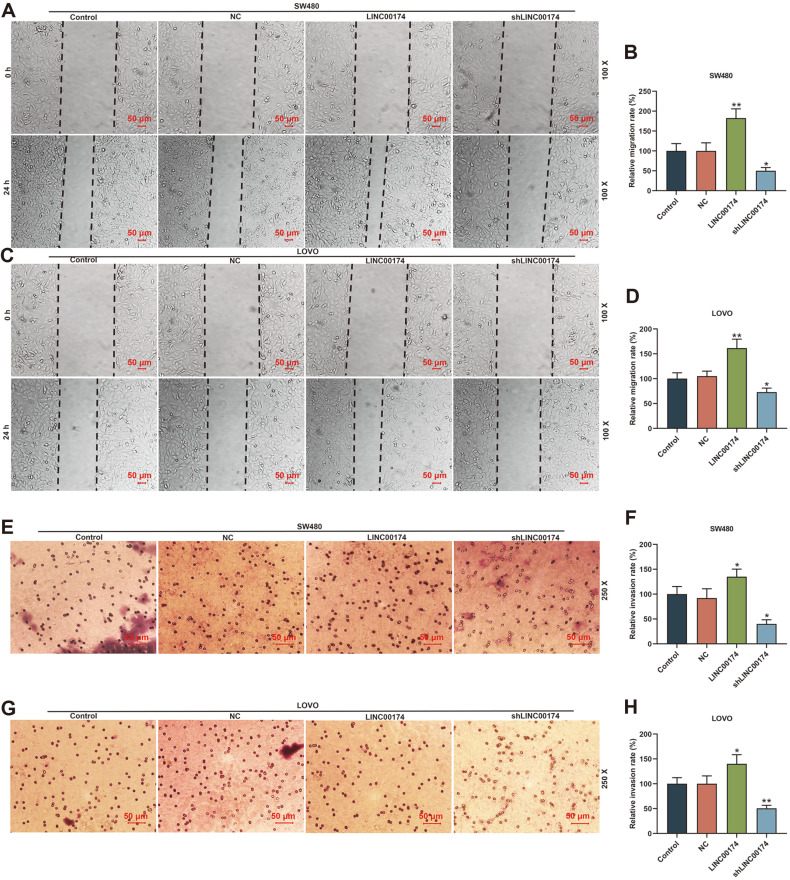
LINC00174 regulated the migration and invasion of colorectal cancer (CRC) cells. (**A-D**) The migration distance of cells in the CRC cell lines of the Control, NC, LINC00174, shLINC00174 groups was determined by the wound healing assay (Magnification×100). (**E-H**) Overexpression of LINC00174 promoted cell invasion; LINC00174 knockdown had the opposite effect, detected by Transwell experiment (Magnification × 250). *p < 0.05, **p < 0.01 vs NC.

To understand the impact of LINC00174 on the biological characteristics of CRC, we transfected LINC00174 overexpression plasmids and shLINC00174 into SW480 cells or LOVO cells. As we wished, LINC00174 was successfully overexpressed or reduced (*p* < 0.05, Figs. 2B and 2C). Subsequently, we used functional experiments to explore the role of LINC00174. The cell viability of the LINC00174 group was dramatically increased, while shLINC00174 had the effect of inhibiting cell viability (*p* < 0.05, Figs. 2D and 2E). Similarly, overexpression of LINC00174 increased the number of cell clones, while knocking out LINC00174 decreased the number of cell clones (*p* < 0.01, Figs. 2F-2I). Moreover, as shown in Figs. 3A-3H, the cell migration rate and invasion rate of CRC were incredibly increased in the LINC00174 group and markedly decreased in the shLINC00174 group (*p* < 0.05, Figs. 3A-3H).

### LINC00174 May Target miR-3127-5p to Participate in CRC Progression

**Fig. 4:**
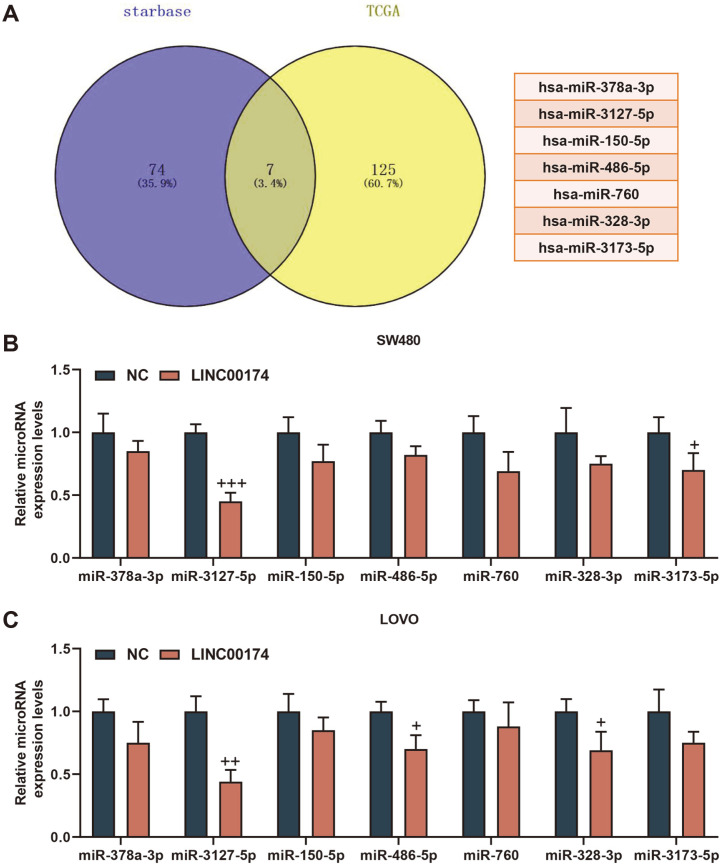
MiR-3127-5p may be a potential therapeutic target in colorectal cancer (CRC). (**A**) The starBase website was used to predict the miRNAs that LINC00174 may bind to, and the TCGA database (http://cancergenome.nih.gov/) was used to obtain miRNAs that were lowly expressed in rectal adenocarcinoma, using the Venny 2.1.0 website to obtain the intersection. (**B-C**) The RT-qPCR was used to determine the expression of target miRNAs in SW480 and LOVO cells. ^+^*p* < 0.05, ^++^*p* < 0.01, +++*p* < 0.001 vs NC.

**Fig. 5:**
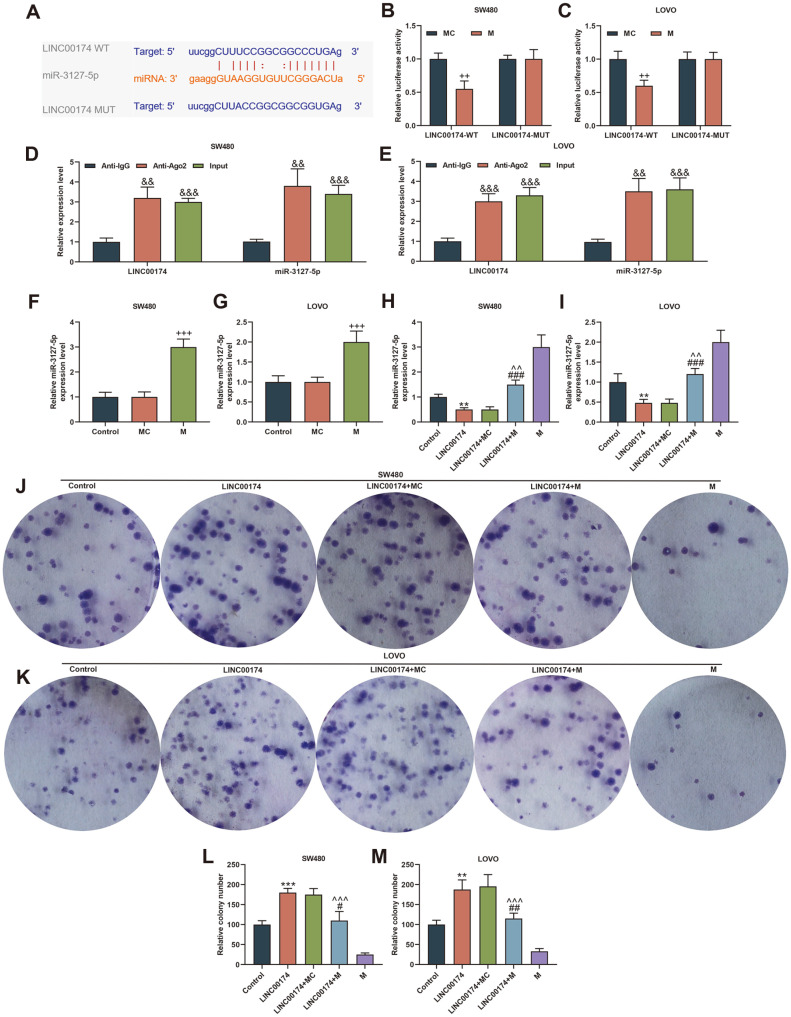
LINC00174 affected the proliferation of colorectal cancer (CRC) cells through miR-3127-5p. (**A**) The binding site of LINC00174 and miR-3127-5p was predicted using the starBase website. (**B-E**) Dual-luciferase report analysis and RNA immunoprecipitation verified the targeting relationship between LINC00174 and miR-3127-5p. (**F-G**) The transfection rate of miR-3127-5p mimic transfected SW480 or LOVO cells was detected by RT-qPCR. U6 was internal reference. (**H-I**) The expression of miR-3127-5p in Control, LINC00174, LINC00174+miR-3127-5p mimic control, LINC00174+miR-3127-5p mimic, miR-3127-5p mimic group was detected by RT-qPCR. U6 was internal reference. (**J-M**) Clone formation experiments were used to detect the proliferation of SW480 or LOVO cells in each group. ^++^*p* < 0.01, ^+++^*p* < 0.001 vs miR-3127-5p mimic control (MC); ^&&^*p* < 0.01, ^&&&^*p* < 0.001 vs Anti-lgG; **p < 0.01, ***p < 0.001 vs Control; ^#^*p* < 0.05, ^##^*p* < 0.01, ^###^*p* < 0.001 vs LINC00174; ^^*p* < 0.01, ^^^*p* < 0.001 vs M.

In order to explore the molecular mechanism of LINC00174 functioning, we predicted 81 possible miRNAs bound by LINC00174 through the starBase website, and obtained 132 miRNAs with low expression in rectal adenocarcinoma through the TCGA database. Subsequently, we used the Venny 2.1.0 website to take the intersection of the miRNAs obtained from the two databases, and finally obtained 7 miRNAs, namely hsa-miR-378a-3p, hsa-miR-3127-5p, hsa-miR-150-5p, hsa-miR-486-5p, hsa-miR-760, hsa-miR-328-3p, hsa-miR-3173-5p (Fig. 4A). Moreover, miR-3127-5p was found to be most lowly expressed in SW480 and LOVO cells (*p* < 0.05 Figs. 4B and 4C). Fig. 5A showed miR-3127-5p was targeted by LINC00174. Next, we verified by dual luciferase analysis and RIP assay that LINC00174 could bind to miR-3127-5p (*p* < 0.01, Figs. 5B-5E).

### LINC00174 Affected the Biological Characteristics of CRC Cells through miR-3127-5p

**Fig. 6:**
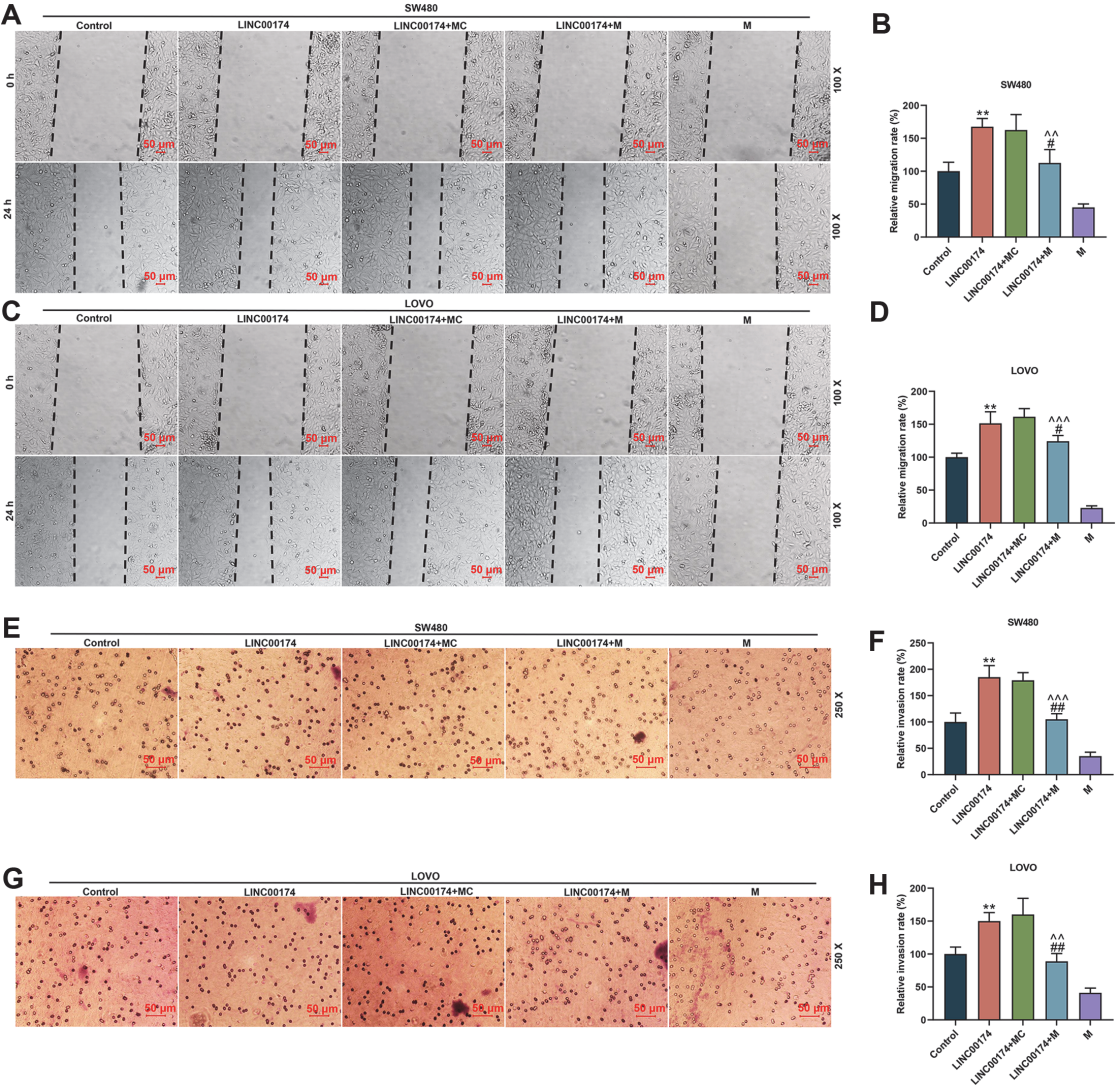
LINC00174 regulated the migration and invasion of colorectal cancer (CRC) cells by targeting miR- 3127-5p. (**A-D**) The cell migration distances in the CRC cell lines of Control, LINC00174, LINC00174+miR-3127-5p mimic control, LINC00174+miR-3127-5p mimic, miR-3127-5p mimic groups were determined by wound healing assay (Magnification × 100). (**E-H**) Transwell experiment was used to detect the invasion rate of CRC cells in each group (Magnification × 250). **p < 0.01 vs Control; ^#^*p* < 0.05, ^##^ *p* < 0.01 vs LINC00174; ^^*p* < 0.01, ^^^*p* < 0.001 vs M.

Whether LINC00174 regulates the function of CRC cells through miR-3127-5p, we have done the following experiments to verify this. First, we tested the transfection rate of miR-3127-5p mimic and showed that miR-3127-5p mimic was successfully transfected into SW480 cells or LOVO cells (*p* < 0.001, Figs. 5F and 5G). Next, miR-3127-5p expression was substantially reduced in the LINC00174 group, and overexpression of LINC00174 could clearly reduce miR-3127-5p mimic-induced miR-3127-5p levels (*p* < 0.01, Figs. 5H and 5I). Third, we also found that miR-3127-5p mimic partially offset the effect of LINC00174 on cells, shown that inhibited cell proliferation, migration and invasion (*p* < 0.05, Figs. 5J-5M, 6A, 6A-6H).

### LINC00174 Regulated the EMT-Related Protein of CRC Cells through miR-3127-5p/E2F7 Axis

**Fig. 7:**
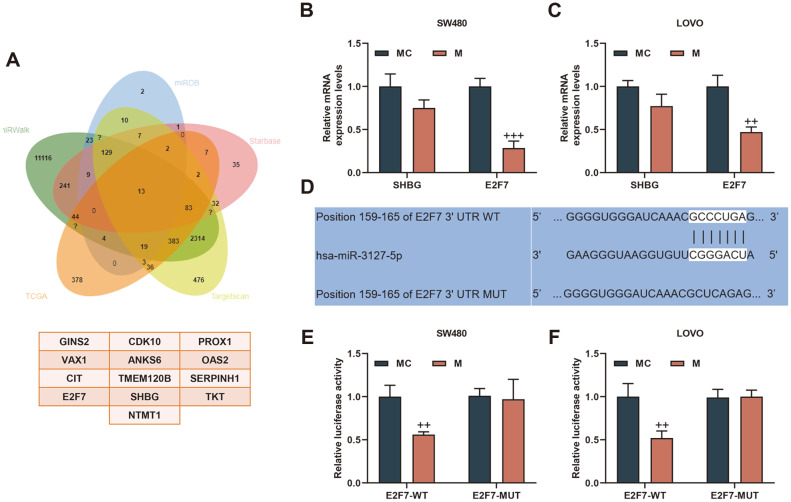
E2F7 was a target gene of miR-3127-5p. (**A**) The miRWalk (http://mirwalk.umm.uni-heidelberg.de/), miRDB (http://mirdb.org/), starBase, Targetscan (http://www.targetscan.org/vert_72/docs/help.html) were used to predict miR-3127- 5p target genes. The Cancer Genome Atlas (TCGA) database (http://cancergenome.nih.gov/) analyzed mRNAs that are abnormal expressed in rectal adenocarcinoma. Venn diagram was used to analyze the intersection of the data generated from the databases. (**B-C**) SHBG and E2F7 expressions were detected by RT-qPCR. (**D-F**) E2F7 bound to miR-3127-5p as predicted and verified by Targetscan and dual luciferase assay. ^++^*p* < 0.01, ^+++^*p* < 0.001 vs MC.

**Fig. 8:**
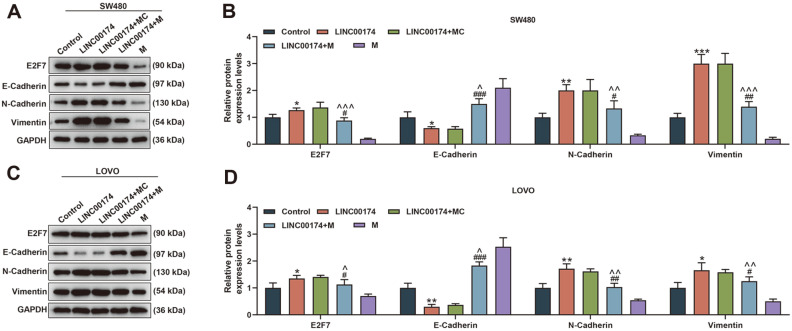
LINC00174 regulated the EMT-related protein of CRC cells through miR-3127-5p/E2F7 axis. (**A-D**) Western blot was used to detect the effects of LINC00174 and miR-3127-5p on the expressions of E2F7, E-Cadherin, NCadherin, and Vimentin in colorectal cancer (CRC) cells. *p < 0.05, **p < 0.01, ***p < 0.001 vs Control; ^#^*p* < 0.05, ^##^*p* < 0.01, ^###^*p* < 0.001 vs LINC00174; ^*p* < 0.05, ^^*p* < 0.01, ^^^*p* < 0.001 vs M.

In addition, we used miRWalk, miRDB, Starbase, Targetscan to predict miR-3127-5p target genes; and crossed the TCGA database with mRNA highly expressed in colorectal adenocarcinoma. As a result, 13 common mRNAs were obtained: GINS2, CDK10, PROX1, VAX1, ANKS6, OAS2, CIT, TMEM120B, SERPINH1, E2F7, SHBG, TKT, and NTMT1 (Fig. 7A). Among them, we selected the top two genes in the context and score in Targetscan: SHBG and E2F7 for the next step. E2F7 expression was suppressed in miR-3127-5p mimic group (*p* < 0.01, Figs. 7B and 7C). Besides, E2F7 bound to miR-3127-5p as predicted and verified by Targetscan and dual luciferase assay (*p* < 0.01, Figs. 7D-7F). Moreover, it was found that LINC00174 overexpression up-regulated the expressions of N-Cadherin, Vimentin, E2F7, and down-regulated the expression of E-Cadherin; however, miR-3127-5p mimic partially reversed the regulation of LINC00174 overexpression (*p* < 0.05, Figs. 8A-8D).

## Discussion

LncRNAs play various roles in the progression of CRC [18, 25]. LncRNA MALAT1 may promote the biological characteristics of CRC through its target protein AKAP-9 [26]. Studies by Zhu *et al*. showed that high expression of MALAT1 can promote the development of CRC by regulating miR-145/SOX9 axis [27], suggesting that the same lncRNA has multiple regulatory mechanisms involved in tumor progression.

LINC00174 plays a pro-cancer role by regulating different functions of cancer cells. In glioma, LINC00174 accelerated carcinogenesis of glioma by promoting cell proliferation, migration, invasion and glycolysis of glioma cells [10, 12]; moreover, LINC00174 down-regulation decreases chemoresistance to temozolomide in human glioma cells [11]. In laryngeal papilloma, LINC00174 enhanced the proliferation and apoptosis evasion of laryngeal papilloma cells by regulating miR-4500/BZW2 axis [15]. LINC00174 involved in cell migration and lipid metabolism, and is a novel prognostic factor in thymic epithelial tumors [14]. It has been reported that LINC00174 can be used as the ceRNA of miR-1910-3p, regulating the target gene TAZ to facilitate the progress of CRC [16], suggesting that LINC00174 can be used as a new drug therapy target for CRC. In this study, LINC00174 was up-regulated in CRC clinical samples and cells, and the abnormal expression of LINC00174 was significantly associated with poor prognosis in patients with CRC. In cell experiments, we found that LINC00174 promoted CRC cell viability, which is consistent with previous studies [16]. However, the difference is that heterologous overexpression of LINC00174 also accelerated the migration and invasion ability of CRC cells, and may promote EMT protein and E2F7 by competitively binding miR-3127-5p to play a cancer-promoting role in CRC. In addition, some scholars have studied the role of LINC00174 in other diseases [10, 11, 28]. Our experimental results are consistent with the report by Wang *et al*.; they found that knocking down LINC00174 inhibited the proliferation of glioblastoma cells and were also associated with tumor grade in glioblastoma patients [29]. In hepatocellular carcinoma, LINC00174 was significantly up-regulated and promoted the malignant phenotype of hepatocellular carcinoma cells by regulating S100A10 as a sponge of miR-320 [30].

More and more studies have shown that lncRNA can affect the binding of miRNA and its target gene by binding to the miRNA site to regulate the expression of the target gene, which is the ceRNAs regulatory network [31]. For example, LncRNA UICLM as a ceRNA of miR-215 regulates the expression of ZEB2, thereby promoting the proliferation, invasion, EMT and stem cell characteristic functions of CRC, and promoting liver metastasis of CRC [19]. Similarly, we propose to assume that LINC00174 can be used as a "molecular sponge", blocking miR-3127-5p’s post-transcriptional inhibition of downstream target genes, so that the function of target genes can be restored. Based on bioinformatics analysis, we examined the effects of LINC00174 and miR-3127-5p on the expression of E2F7, and found that LINC00174 can regulate the expression of E2F7 through miR-3127-5p in CRC cells. Interestingly, E2F7 has been shown to be highly expressed in CRC and has the promoting effect on the malignant biological function of CRC [32]. Of course, more downstream regulatory networks require further experimentation.

MiRNA plays a momentous role in the progression of CRC by up-regulating oncogenes or down-regulating oncogenes [33]. For example, the latest research found that miR-185-3p is down-regulated in CRC, and up-regulating miR-185-3p can enhance the chemotherapy sensitivity of CRC cells by targeting AQP5 [34]. Ma *et al*. found that knocking down LINC02163 attenuated CRC cell proliferation and metastasis through the miR-511-3p/AKT3 axis [35]. MiR-3127-5p is the mature miR-3127 derived from miR-3127 during maturation. Yu *et al*. pointed out that miR-3127-5p was a target miRNA by SNHG1 to suppress the anti-cancer roles of baicalein in cervical cancer [36]. To our knowledge, though the latest research shows that miR-3127-5p is differentially expressed in CRC, this is the first report of the effect of miR-3127-5p in CRC. It was found that miR-3127-5p may function as a tumor suppressor gene in CRC, revealing that miR-3127-5p may be a potential treatment target for CRC. In addition, whether LINC00174 will develop normal cells into cancer cells is need to further study.

In summary, the expression of LINC00174 is up-regulated and the expression of miR-3127-5p is down-regulated in CRC cells. Intervention of LINC00174 expression can play a role in promoting the malignant phenotype and EMT of CRC cells through miR-3127-5p/E2F7 axis. This study provides an experimental basis for the application of LINC00174 and miR-3127-5p in the diagnosis and molecular targeting of CRC. The next step will continue to explore the impact of LINC00174/miR-3127-5p/ E2F7 axis on CRC in vivo.

## Figures and Tables

**Table 1:** The correlation between the expression of LINC00174 and clinical characteristics of patients with colorectal cancer.

Clinicopathlogical features	LINC00174 expression		*p* value

	Low (%)	High (%)	
Gender			
Male	24(54.5)	20(45.5)	0.875
Female	19(52.8)	17(47.2)	
Age			
< 60	15(62.5)	9(37.5)	0.304
≥ 60	28(50)	28(50)	
Tumor size (cm)			
< 4	24(77.4)	7(22.6)	<0.001
≥ 4	19(38.8)	30(61.2)	
Lymphovascular invasion			
Absent	35(66.0)	18(34.0)	0.002
Present	8(29.6)	19(70.4)	
Differentiation			
Well-moderate	22(46.8)	25(53.2)	0.137
Poor	21(63.6)	12(36.4)	
Depth of invasion			
T1+T2	23(75.8)	8(24.2)	0.004
T3+T4	20(46.8)	29(53.2)	
Lymph node metastasis			
N0	25(73.5)	9(26.5)	0.002
N1-N2	18(39.1)	28(60.1)	
Distant metastasis			
M0	33(63.5)	19(36.5)	0.0176
M1	10(35.7)	18(64.3)	
TNM stage			
Ⅰ+Ⅱ	25(69.4)	11(30.6)	0.011
Ⅲ+Ⅳ	18(40.9)	26(59.1)	
